# Biotechnological Potential of the *Fusarium fujikuroi* Complex From Amazonian *Oenocarpus bacaba*: Lipase Production and Characterization

**DOI:** 10.1002/jobm.70067

**Published:** 2025-06-08

**Authors:** Lucely Nogueira dos Santos, Rafael Firmani Perna, Ana Carolina Vieira, Kalebe Ferreira Furtado, Alex Fernando de Almeida, Cristiane Angélica Ottoni, Leandro Mantovani de Castro, Felipe Mello, Yoannis Domínguez, Nelson Rosa Ferreira

**Affiliations:** ^1^ Laboratory of Biotechnological Processes (LAPROBIO), Institute of Technology Federal University of Pará Belém Pará Brazil; ^2^ Institute of Science and Technology Federal University of Alfenas; ^3^ Federal University of Tocantins; ^4^ Institute of Biosciences São Paulo State University

**Keywords:** filamentous fungi, Fusarium, lipases, morphology, phylogenetics

## Abstract

This study investigates the diversity of filamentous fungi in the Amazon biome, particularly the genus *Fusarium*, known for producing biotechnologically valuable metabolites. The research aimed to isolate and identify fungi from *Oenocarpus bacaba* Mart. fruits and analyze the biochemical properties of lipase produced by the strains (maximum activity of 1750 U mL⁻¹). Two isolates, FF1 and FF2, were identified using morphological and molecular techniques, with ITS sequence data suggesting they belong to the *Fusarium fujikuroi* species complex, though the exact species remains unconfirmed. This is the first report that highlights the biotechnological potential of the *F. fujikuroi* complex isolated from *Oenocarpus bacaba*, emphasizing the relevance of Amazonian biodiversity as a source of microorganisms with promising applications in sustainable industrial processes. The results show that native fruits, such as bacaba, are effective matrices for prospecting filamentous fungi producers of enzymes of biotechnological interest, such as lipases. These findings reinforce the importance of rational exploitation of the Amazonian microbiota for the development of bioproducts and eco‐efficient industrial processes.

**Abbreviations**
BLAST: basic local alignment search tool
CCRD: central composite rotational design
FF: filamentous fungi
IB: Bayesian Inference
ITS: internal transcribed spacers
NCBI: national center for biotechnology information
nrDNA: region of the nuclear ribosomal DNA
PCR: polymerase chain reaction
PDA: potato dextrose agar
pH: X1
SDA: Sabouraud dextrose agar
SisGen: associates of biodiversity resources
SL: soluble lipase
temperature: X2

## 1 Introduction

*Oenocarpus bacaba* Mart. (Arecaceae), a palm native to the Amazon rainforest, produces fruits with high nutritional value, rich in bioactive compounds (e.g., flavonoids, anthocyanins), antioxidants, and unsaturated fatty acids [1, 2]. The fruit is widely consumed as a nutritious and flavorful drink in the Amazon [3]. Research in the industrial sector focuses on extracting its energy components, lipids, and residual oils, highlighting its potential for both food and industrial applications [4].

The Amazon region is a critical hotspot for microbial diversity. The isolation and identification of new fungal species highlight systemic gaps in our knowledge, including geographic disparities in sampling and research. Preserving and characterizing fungi from underrepresented regions is vital for protecting fungal diversity, ecosystem functions, agricultural security, and human health [5]. Organisms from this biome are highly valuable for their potential to yield new bioactive compounds, support sustainable production, and provide genomic insights, paving the way for discoveries [6].

Filamentous fungi (FF) are present in several ecosystems and stand out for their biotechnological potential in the synthesis of various bioactive compounds of interest to man and the environment [7, 8]. The isolation of FF from fruits of the Amazon biome leads to the discovery of several biomolecules, such as enzymes, which are important in various industrial processes [2]. The development of bioprocesses and the search for FF that produce enzymes efficiently represent strong technological demands. Lipids, for example, have been successfully applied in a wide variety of industrial sectors, from food and beverages, animal feed, cleaning products, medicines, cosmetics, waste treatment to biofuels [9]. This study brings for the first time the study of the biotechnological potential of FF isolates from Amazon region, through optimization for temperature and pH for the hydrolysis of olive oil by reaction catalyzed by the soluble lipase (SL) produced by the fungal isolates.

The choice of *Oenocarpus bacaba* as microbial matrix is justified by its association with a diverse and functionally active fungal microbiota, characteristic of the Amazonian biome. Despite the growing scientific interest in prospecting for Amazonian microorganisms, there is still a lack of studies specifically focused on the filamentous fungi associated with bacaba [10], which reinforces the relevance and originality of research in this area. Previous studies have shown that microorganisms isolated from this region, such as *Aspergillus* sp., are able to produce bioactive metabolites such as kojic acid, reinforcing the biotechnological potential of Amazonian fungi [2]. In this context, native fruits such as bacaba stand out as promising sources for the prospecting of filamentous fungi with ability to synthesize enzymes of industrial interest, such as lipases. As observed by Batista et al. [10], about 41% of the isolates from fungal strains isolated from *O. bacaba* showed lipolytic activity, evidencing the technological value of this biological resource. These findings highlight the importance of rational exploration of the Amazon microbiota for the discovery of new biocatalysts and the development of eco‐efficient industrial processes.

*Fusarium* is a genus of FF in the phylum Ascomycota, characterized by species with dark blue to black perithecia, clavate asci, and ellipsoidal to cylindrical ascospores. As reported by Todorović et al. [11] and Ajaml et al. [12], species of the genus *Fusarium* are capable of producing ascospores through sexual reproduction, along with three distinct types of asexual spores: macroconidia, microconidia, and chlamydospores. Nonetheless, sexual reproduction is relatively rare, occurring in fewer than 20% of described species. The genus exhibits significant morphological, physiological, and ecological diversity, contributing to its complex and challenging taxonomy [13]. Species of *Fusarium* are recognized as promising sources of several enzymes for industrial application, such as, a l‐glutaminase [14], exoglucanase and endoglucanase [15], lipase [16] in the production of pigments [17], nitrogen containing compounds, polyketides, terpenoids, steroids, and phenolics, presented activities antibacterial, antifungal, phytotoxic, antimalarial, anti‐inflammatory, and cytotoxic activities [18].

In a broad concept, *Fusarium* species are grouped into several species complexes [13, 19]. Species within each complex share very similar macroscopic and microscopic characteristics, making their identification through phenotypic methods challenging. The taxonomic system based on the morphology of *Fusarium* is inadequate and detection and identification procedures are time‐consuming and error prone. So, the association of molecular data information with morphological identification is important to accurately identify fusariform fungi [20]. Crous et al. [13] proposed a workflow for the identification and characterization of these organisms that includes analysis of macromorphological and micromorphological characteristics and identification by molecular markers such as internal transcribed spacers (ITS) region of the nuclear ribosomal DNA (nrDNA) and comparison with reference ITS sequences in databases.

Therefore, research isolating new species in highly diverse regions like the Amazon rainforest, combining morphological and molecular techniques, is crucial for identifying microorganisms with industrial potential, such as enzyme producing fungi. This study aimed to isolate and identify fungal strains from the fruits of *Oenocarpus bacaba* using both morphological and molecular techniques, and to biochemically characterize the soluble lipase produced by these isolates.

## 2 Materials and Methods

### 2.1 Collection of Samples and Isolation of Microorganisms

The fruits of *Oenocarpus bacaba* were harvested in the Bosque Menino Jesus Community, in the Cametá city, Pará, Brazil (2.15′17.0″ S, 49.23 23′47.9″ W). This study was registered in the Brazilian National System of Management and Access to Genetic Heritage and Traditional Knowledge Associates of biodiversity resources ‐ SisGen, in compliance with Brazilian Law n° 13.123/2015 and its regulation, under registration number AAC153B. The research involved solely plant material from a native species widely distributed in the Amazon, without access to traditional knowledge or resources exclusive to the Bosque Menino Jesus community.

The bacaba fruits were washed with sterile water in a ratio of 1:1 (w/v), then softened using sterile water at ± 39°C in a ratio of 300 g of fruit to 700 mL of water for 15 min, to facilitate the separation of the pulp from the stone. The separation was carried out manually, and the bacaba pulp was transferred to a sterile Erlenmeyer flask, sealed and kept in a moist chamber at approximately 25°C, under aerobic conditions and relative humidity between 80% and 85%, for a period of 20 days, to stimulate the growth of fungal mycelia through natural fermentation. To obtain the fungal colonies, the fungal mycelia that developed during the fermentation period were inoculated onto Sabouraud Dextrose Agar, with the pH adjusted to ~5.6 with HCl, plus chloramphenicol (100 mg/L) to prevent bacterial growth. The plates were incubated at 28 ± 1°C for 5 days. From the emergence of fungal colonies, successive subcultures were carried out to obtain pure cultures that were tested for lipase enzyme activity [10].

### 2.2 Morphological and Molecular Identification of *Fusarium* Species

The macromorphological identification was performed using two filamentous fungi coded: FF1 and FF2. The fungal isolates were cultivated in Petri dishes (90 mm) containing potato dextrose agar (PDA) and incubated at 25 ± 1°C for 10 days. The diameter and color of the surface of the colony and the mycelium of the substrate (back/bottom of the agar plate) were recorded [21]. For microscopic examination, each isolate was subcultured in blocks of malt extract agar (MEA, 2%) of approximately 1 cm^2^ containing a fragment (0.5 cm^2^) [22]. The agar blocks were inserted into a sterile microscope slide covered with a sterile coverslip and incubated in a petri dish (90 mm). The culture was maintained under controlled conditions of temperature (30 ± 1°C) and illumination (photon flux of approximately 70 µmol of photon m‐2 s^−1^, with a photoperiod of 12:12 h (light:dark) for ten days. For microscopic observation, a drop of cotton blue lactophenol was applied to the fungal mycelium in the coverslip. The coverslip was placed face down on a microscope blade [21]. The morphology of microconidia, macroconidia, conidiogenous cells (phials) and chlamydospores were observed, according to the criteria proposed by Aparecido and Rosa [23].

Five 6 mm plugs of the FF1 or FF2, previously grown in a Petri dish containing PDA were inserted into a 250 mL Erlenmeyer flask filled with 50 mL of culture medium (composition in g L^−1^): malt extract (3.0), glucose (10.0), yeast extract (3.0), and bacteriological peptone (5.0). The Erlenmeyer flask was incubated at 150 rpm, 30°C ± 1 for 72 h. About 0.05 g of fungal biomass was transferred to a sterile microtube containing 100 μL of EAR buffer (TRIS/HCl: 100 mM, pH 8.5; EDTA: 5 mM, pH 8.0; 200 mM NaCl and 0.2% SDS) and 8 μL of protease K (20 mg. mL^−1^). The sample was incubated at 55°C under agitation of 300 rpm “overnight”. At the end of the period, the sample was heated for 10 min to inactivate proteinase K and then centrifuged at 12,000 rpm for 5 min at 4°C. After this stage, 500 μL of TE buffer (TRIS/HCl: 10 mM, pH 7.5 and EDTA: 1 mM) and 5 μL of RNAse A (3 mg. mL^−1^) were added and incubated for another 10 min at 37 ± 1°C. The genomic DNA sample was stored at ‐20°C.

The amplification of the nrDNA transcribed internal spacers (ITS) region was performed by PCR using the primers pair ITS5 (5′‐TCCGTAGGTGAACCTGCGG‐3′) ITS4 (5′‐TCCTCCGCTTATTGATATGC‐3′), which targets the entire ITS region and generates an expected fragment size of approximately 550‐600 [24]. The amplification mix was composed of 1 μL of the target DNA, 0.2 μL of Taq DNA Polymerase GE Healthcare™, 0.5 μL of dNTP MIX Promega Corporation™, 100 mol μL^−1^ of each primer, 2,5 μL of 10x PCR Buffer GE Healthcare™ and l8 μL of MilliQ water, with a final volume of 25 μL.

The polymerase chain reaction (PCR) reaction was performed in a thermal cycler with the following conditions: initial denaturation at 95°C for 5 min, followed by 35 cycles of 30 s at 94°C, 30 s at 52°C with a final extension at 72°C for 1 min. After the last cycle, the temperature was maintained at 72°C for 8 min to ensure that any remaining DNA was completely elongated. Finally, the reaction chamber was cooled to 4°C indefinitely for short‐term storage of the DNA fragments obtained.

The amplification products were cleaned using Purification PureLink™ PCR Kit, checked for quality and concentration by 1% agarose gel electrophoresis. Subsequently, the fragments were sequenced using the same PCR conditions at the Human Genome and Stem Cell Research Center, Institute of Biosciences, the University of São Paulo.

The sequences obtained for each sample (forward and reverse) were visualized and aligned in BioEdit v. 7.0.9 [25], to generate the consensus sequences. Each consensus sequence was 558 bp for FF1 and 557 bp for FF2. They were deposited in the GenBank® database [26], of the National Center for Biotechnology Information (NCBI) (FF1, PP401976; FF2, PP401977) and compared with available nucleotide sequences in the rRNA/ITS database using BLAST® (Basic local Alignment search tool) and limiting the search to ITS sequences of fungi type specimens and reference materials. The samples were identified as belonging to the genus *Fusarium* Link (Ascomycota Caval. Sm.: Hypocreales Lindau) and showed higher identity with species of the *Fusarium fujikuroi* species complex. Therefore, ITS sequences of this species complex and other species of *Fusarium* previously recorded in Brazil [27], available at Genbank database (Table 1) were downloaded for subsequent analyzes. In addition, sequences of the related genera *Cordyceps* Fr. and *Trichoderma* Pers. were obtained (*C. cylindrica* Peth, AJ786558; *C. militaris* (L.) Fr., AF153264; *T. aureoviride* Rifai, NR144877; *T. pseudokoningii* Rifai, NR120296) to be used as outgroup. The set of sequences was aligned using MAFFT v. 7.110 [28] with the option DPPartTree.

**Table 1:** Species of the genus *Fusarium* used in the ITS phylogenetic analysis, Genbank accession numbers and type of material.

Species	Accession number	Material
*Fusarium acutatum* Nirenberg & O'Donnell	NR111142	Type
*Fusarium annulatum* Bugnic.	NR138275	Type
*Fusarium awaxy* Petters Vandresen, Galli‐Terasawa, Terasawa & Glienke	MH252921	Reference
*Fusarium bactridioides* Wollenw.	NR120262	Type
*Fusarium begoniae* Nirenberg & O'Donnell	NR111864	Type
*Fusarium bulbicola* Nirenberg & O'Donnell	NR152942	Type
*Fusarium chlamydosporum* Wollenw. & Reinking	NR172283	Type
*Fusarium concentricum* Nirenberg & O'Donnell	NR111886	Type
*Fusarium denticulatum* Nirenberg & O'Donnell	NR138359	Type
*Fusarium dlaminii* Marasas, P.E. Nelson & Toussoun	NR182425	Type
*Fusarium ficicrescens* Al‐Hatmi, Mirab., Stielow & de Hoog	NR152915	Type
*Fusarium fujikuroi* Nirenberg	NR111889	Type
*Fusarium guttiforme* Nirenberg & O'Donnell	NR120264	Type
*Fusarium lactis* Pirotta	NR111887	Type
*Fusarium lateritium* Nees	AF310978	Reference
*Fusarium merismoides* Corda	KF381089	Reference
*Fusarium napiforme* Marasas, P.E. Nelson & Rabie	NR149349	Type
*Fusarium nygamai* L.W. Burgess & Trimboli	NR130698	Type
*Fusarium phyllophilum* Nirenberg & O'Donnell	NR182426	Type
*Fusarium pseudonygamai* O'Donnell & Nirenberg	NR137162	Type
*Fusarium ramigerum* Nirenberg & O'Donnell	NR111888	Type
*Fusarium sacchari* (E.J. Butler & Hafiz Khan) W. Gams	NR174875	Type
*Fusarium solani* (Mart.) Sacc.	NR163531	Type
*Fusarium sterilihyphosum* Britz, Marasas & M.J. Wingf.	KF576628	Reference
*Fusarium subglutinans* (Wollenw. & Reinking) P.E. Nelson, Toussoun & Marasas	NR182424	Type
*Fusarium succisae* J. Schröt. ex Sacc.	NR174876	Type
*Fusarium werrikimbe* J.L. Walsh, L.W. Burgess, E.C.Y. Liew & B.A. Summerell	NR175624	Type

### 2.3 Phylogenetic Analysis

Phylogenetic reconstruction was generated via Bayesian Inference (IB) in MrBayes v. 3.2.7a [29, 30]. The nucleotide substitution model GTR + G (*General time reversible* with gamma distribution), previously identified in jModeltest v. 2.1.10 [31, 32] was selected as the best‐fit model based on the Akaike Information Criterion (AIC) [33]. Two parallel Markov chain Monte Carlo [34] simulations of four chains (were run for 5×10⁠^6^ generations and the convergence of the chains was assessed by the average standard deviation of split frequencies ( < 0.01). One tree was sampled every 1000 trees generated, discarding the first 25% of the trees (burn‐in). The posterior probabilities (PP) were assessed from the 50% majority rule consensus tree calculated from the remaining trees. The estimated sample size (ESS > 200) and the potential scale reduction factor (PSRF = 1.0) were also checked after the runs. Outgroup taxa (*Cordyceps* spp. and *Trichoderma* spp.) were used to root the tree that was visualized and edited in Figtree v. 1.4.4 [35].

### 2.4 Evaluation of the Lipase Production Potential of Isolated Filamentous Fungi

The evaluation of lipase activity by the fungal isolates was established according to the study by Diniz et al. [36], in which the fungal strains were inoculated in a culture medium (composition in g L^−1^): sodium nitrate (0.19), potassium phosphate (0.59), magnesium sulfate heptahydrate (0.25), potassium chloride (0.25), iron (II) sulfate heptahydrate (0.005), glucose (5) and bacteriological agar (15). The medium was enriched with 2% Tween 80 and the pH was adjusted to 6.5. The production activity for lipase was confirmed by the formation of transparent regions around the colonies, indicating the complete fatty acid degradation.

### 2.5 Microbial Culture and Production of Soluble Lipase (SL)

The submerged cell cultivation occurred according to the methodology adapted from Almeida et al. [37], in a synthetic medium, pH 5.5, composed of: 5.5 g L^−1^ of K_2_HPO_4_; 15 g L^−1^ of KHPO_4_; 0.5 g L^−1^ of MgSO_4_.7H_2_O; 1.0% (m v^−1^) of olive oil and 0.2% of yeast extract (m v^−1^). The cultivation was conducted in an orbital shaker at 30°C and 200 rpm for 168 h, inoculating 500 µL of a suspension of 10^7^ spores mL^−1^ of the fungus in Erlenmeyer flasks with 50 mL of sterile culture medium. After cultivation, the total content of the flasks was vacuum filtered (cellulose ester membrane filter, 0.45 µm) and the permeate, containing the SL, was used for the hydrolysis of olive oil.

### 2.6 Experimental Design

An experimental design of the type Central Composite Rotational Design was applied for the optimization of the enzymatic reaction of olive oil hydrolysis. A total of 11 assays were performed, consisting in 4 factorial points, 4 axial points and 3 repetitions at the central point for the study of the factors: pH (X_1_) and temperature (X_2_). The levels were defined according to preliminary experimental results. As response variable (Y_1_), the hydrolytic activity of soluble lipase (U mL^−1^) was obtained (Table 2). The experimental matrix and statistical analysis (Analysis of Variance ‐ ANOVA) were elaborated with the aid of the software Protimiza®, having their prediction models and their response surfaces calculated for a confidence interval of 95% (*p*‐value < 0.05).

**Table 2:** Matrix of the Central Composite Rotational Design of 11 assays for the response hydrolytic activity of the microbial soluble lipase with the respective real and coded values.

Assay	pH (X_1_)	Temperature (°C) (X_2_)	Hydrolytic activity (U mL^−1^) (Y_1_)
1	5.0 (−1)	30 (−1)	1450
2	7.0 ( + 1)	30 (−1)	1200
3	5.0 (−1)	40 ( + 1)	1250
4	7.0 ( + 1)	40 ( + 1)	1500
5	5.59 (−1.41)	35 (0)	1450
6	7.41( + 1.41)	35 (0)	1450
7	6.0 (0)	27.93 (−1.41)	1400
8	6.0 (0)	42.07 ( + 1.41)	1450
9	6.0 (0)	35 (0)	1750
10	6.0 (0)	35 (0)	1650
11	6.0 (0)	35 (0)	1650

### 2.7 Hydrolytic Activity of Olive Oil Determination

The hydrolytic activity of extracellular lipase was determined by the method of hydrolysis of olive oil emulsion, as described by Soares et al. [38], with adaptations. The substrate solution was prepared by emulsion with 25 g olive oil and 75 g 3% (m/m), arabic gum solution. The hydrolytic activity was quantified by neutralization titration with NaOH (0.05 mol L^−1^), standardized with potassium biphthalate. As substrate, a 1% (v/v) solution of olive oil was used, and phenolphthalein was used as an indicator. The enzymatic reaction was performed at 37°C and 175 rpm for 5 min in a shaker‐type orbital shaker (Tecnal®, modelo TE‐4200). The value of hydrolytic activity was calculated (Equation 1):

(1) $$\mathrm{AH}=\frac{\left(V_{a}-V_{b}\right).M.1000}{t.X}$$

where: Va, volume of NaOH solution spent on sample titration (mL); Vb, volume of NaOH solution spent on blank titration (mL); M, molarity of solution NaOH; X, amount of enzyme extract used (mL); t, reactivity time (min).

One unit of hydrolytic activity (U) is defined as the amount of enzyme that releases 1 μmol of fatty acid per minute under specific assay conditions, with activities expressed in U mL⁻¹ [39].

## 3 Results

### 3.1 Morphological Characterization

**Figure 1:**
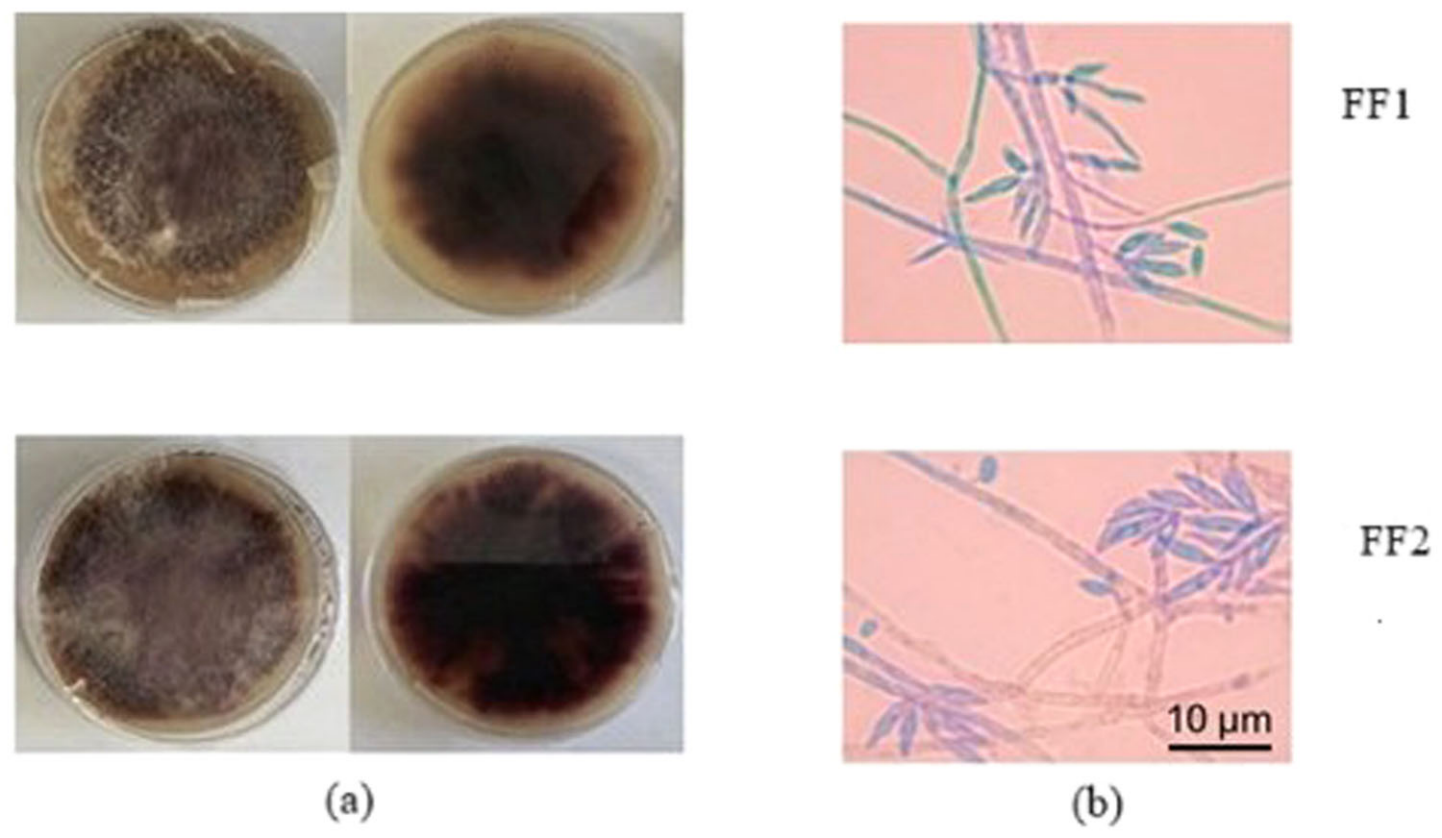
*Fusarium* isolates (FF1 and FF2) from fruits of *Oenocarpus bacaba* from the Amazon rainforest. Macroscopic (a) analysis front and back. Micro‐morphology (b) of macroconidia. Micromorphology of macroconidia observed under a light microscope (scale bar = 10 µm).

The diameters of the colonies were daily measured (FF1, FF2) completely coated the Petri dish on the 5th and 12th day of incubation, respectively. The growth occurred in an irradiated form from the center to the extremities, giving a three‐dimensional aspect. FF1 and FF2 strains exhibited violet and white pigmentation (Figure 1). Colony color varied between vinaceous and violet. Presence of abundant aerial mycelia with a cottony appearance. Microscopic analysis revealed hyaline macroconidia with elongated apical cells, abundant and falcate to straight in shape, containing 3 to 5 septa.

### 3.2 Molecular Characterization

**Figure 2:**
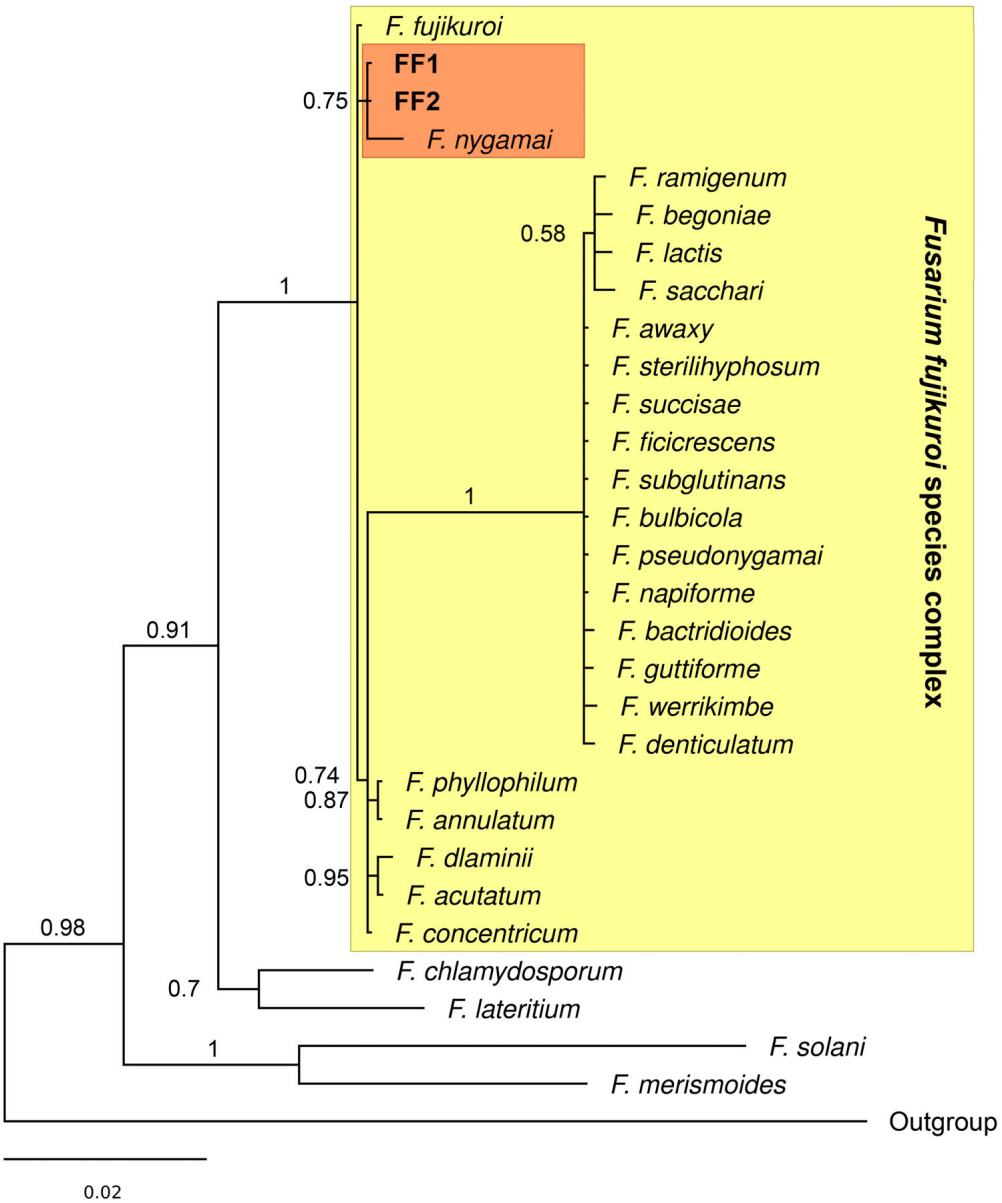
Relationships among *Fusarium* isolates from the bacaba fruits and *Fusarium* species previously recorded in Brazil. 50% majority rule consensus tree based on ITS sequences. The numbers above the branches indicate the posterior probabilities. Outgroup: *T. aureoviride, T. pseudokoningii, C. cylindrica* and *C. militaris*.

The ITS sequences obtained from the isolates FF1 and FF2 are identical, therefore, they likely belong to the same species of *Fusarium*. The Blastn search failed to accurately identify the species since the results showed greater homology with two species of the *Fusarium fujikuroi* species complex: *F. fujikuori* (99.63%) and *F. concentricum* (99.62%). However, the phylogenetic analysis grouped FF1 and FF2 sequences with the rest of the species from this complex included in our data set (Table 1) forming a monophyletic group with the highest posterior probabilities (Figure 2). Thus, FF1 and FF2 also represent a species within the aforementioned complex. Furthermore, FF1 and FF2 form a monophyletic subclade with *F. fujikuroi* and *F. nygamai* (P*p* = 0.75), the latter being the closest species to the samples obtained from bacaba fruits. None of the *Fusarium* species previously recorded in Brazil showed a close relationship with FF1 and FF2, therefore, they may represent a new record for the Brazilian Amazon region.

### 3.3 Optimization of Ph and Temperature Factors By Experimental Design

Table 2 presents the experimental design matrix with their respective responses for the hydrolytic activity of soluble lipase.

For the hydrolytic activity of olive oil to SL, the quadratic terms of the effects of pH and temperature and their interactions were significant to a level of significance of 5% (*p* < 0,05). In accordance with Siegel and Wagner [40], the *F* test applied to verify if variables X explain a significant amount of variation in Y in a regression model.

Table 3 presents the ANOVA for the quadratic model with interaction applied to the hydrolytic activity of olive oil by extracellular lipase. The model showed a high coefficient of determination, indicating a good fit to the experimental data. Additionally, the low pure error confirms the good reproducibility of the responses under the tested conditions.

**Table 3:** Results of the analysis of variance for the quadratic model with interaction for evaluation of the effects of temperature and pH of the hydrolysis of olive oil to soluble lipase.

Source	Sum of squares	Degree of freedom	Men square	*F* value
Model	246089.0	3	82029.7	22.3
Residues	25729.2	7	3675.6	
Lack of Fit	19062.5	5		
Pure Error	6666.7	2		
Total	271818.2	10		
R^2^ = 90.53%	*F* = 3;7;0.05 = 4.35			

For the hydrolytic activity of LS, approximately 90.53% of the variability of the observed responses can be explained by model adjustment (equation 2), which was prepared from the significant variables. The model presented high R^2^ and value of 4.35 for F_tabulated_, lower than the calculated value that was 22.3, we have the value for variation in explained variables greater than the unexplained variation, therefore, the model is considered valid.

(2) $${U\mathrm{ml}}^{-1}=1683.33-138.54{.\left(\mathrm{pH}\right)}_{1}^{2}-151.04\;.{\left(T\right)}_{2}^{2}+125\;{(\mathrm{pH})}_{1}{.(T)}_{2}$$

**Figure 3:**
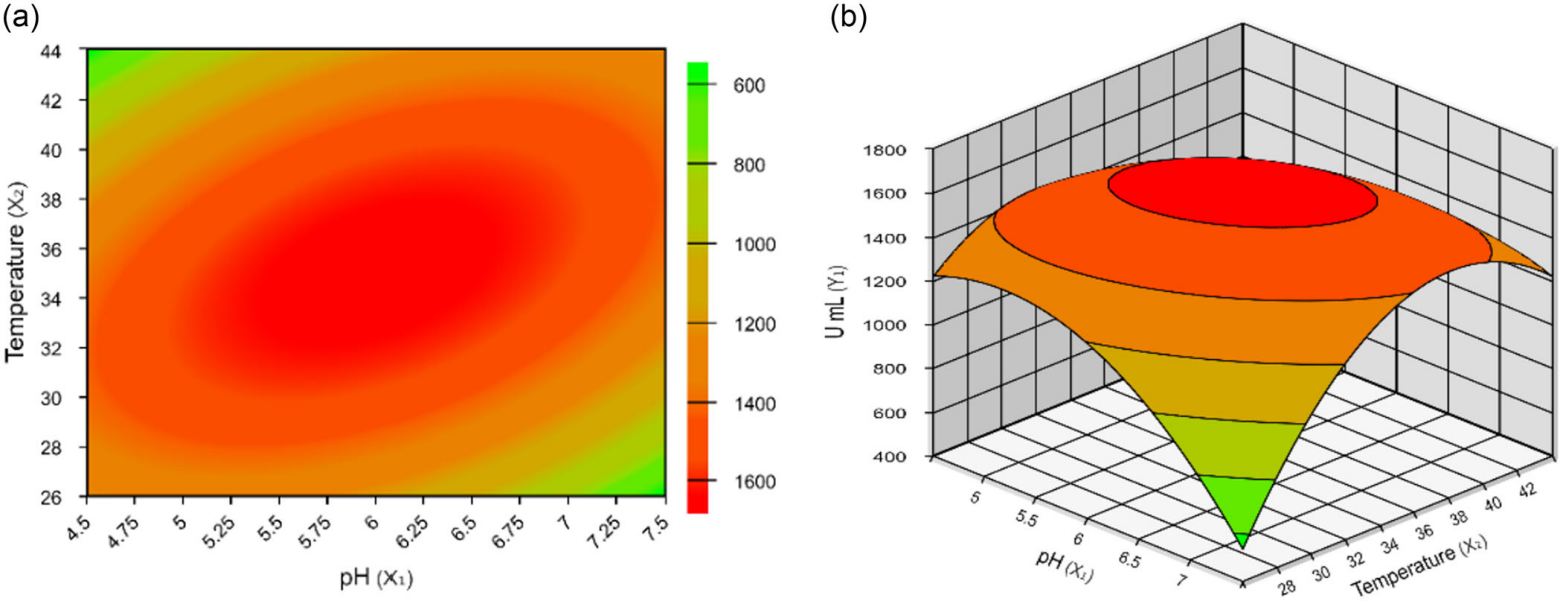
Response surface (a) and contour curves (b) for hydrolytic activity function of pH and temperature of the reaction medium in the hydrolysis of olive oil by the soluble lipase.

The results obtained from the response surface (Figure 3a) and contour curves (Figure 3b) for the hydrolytic activity of olive oil by the SL illustrates the enzyme's behavior when evaluated under varying pH and temperature conditions.

## 4 Discussion

This study aimed to identify novel filamentous fungi from *O. bacaba* fruits in the Brazilian Amazon rainforest. The findings suggest that the two new isolates likely belong to *Fusarium nygamai* or another species within the *Fusarium fujikuroi* complex. This marks the first reported isolation of *Fusarium* from bacaba fruits.

Currently, the genus *Fusarium* comprises at least 300 phylogenetically distinct species, 20 species complexes and nine monotypic lines (species based on the specificity of the host as a ‘forma specialis’ system (f. sp.). Evolutionary selections and anthropogenic activities create constant pressure that encourages changes in species, leading to the development of new strains [41]. Macroscopic and microscopic characteristics, such as colony color, composition, shape and arrangement of the microconidia are important aspects for the differentiation of *Fusarium* species. However, morphological analysis is not always sufficient to characterize cryptic species and the identification by phenotypic techniques of *Fusarium* strains are erroneous in up to 50% of cases [42]. This is due to multiple factors, including morphological characteristics that separate the species, especially the closest ones, besides the high morphological and physiological plasticity observed in *Fusarium* [43]. Therefore, it is important to associate morphological characteristics with molecular biology identification techniques, which assist in the taxonomy characterization and identification of *Fusarium* isolates [44, 45].

*Fusarium* isolates show variability in morphological characteristics, including colony color, mycelial texture, pigmentation, sporulation, branching patterns and size and shape of the conidia [46]. In the present study, the morphological characterization of FF1 and FF2 isolates was based on macroscopic and microscopic characteristics, such as mycelial growth patterns and pigmentation. Both isolates presented white aerial mycelia and produced hyaline macroconidia with elongated apical cells and fusiform shape (Figure 1), characteristics consistent with those described by Harish et al. [46] for species within the *Fusarium fujikuroi* species complex. The isolates showed significant phenotypic diversity, including colors of colonies ranging from violet and pink to white, with pigment diffusion and conidial morphology variable. The presence of fluffy white mycelia and fusiform macroconidia in FF1 and FF2 aligns with a subset of *Fusarium fujikuroi* isolates that exhibited similar characteristics, such as white to off‐white colonies, fluffy aerial growth, and sickle‐shaped or pointed macroconidia. These morphological similarities support the classification of the isolates in the present study within the *F. fujikuroi* species complex.

The molecular techniques applied served as a proxy for identifying the isolates revealing that FF1 and FF2 belong to a clade within the *F. fujikuroi* complex (Figure 2), with *F. nygamai* being the closest species to the samples obtained from bacaba fruit (P*p* = 0.75). However, ITS sequences provided low resolution for distinguishing closely related species within the *F. fujikuroi* species complex (Figure 2). The ITS region is useful for identification at genus level and species complex level but it often lacks sufficient variability to resolve species boundaries [47, 48]. Therefore, multilocus approaches using more informative markers (e.g. tef1, rpb2, acl1, CaM, rpb1 and tub2) are recommended for accurate species identification and taxonomic confirmation in *Fusarium* species [49, 50, 51].

*Fusarium nygamai* is a member of the species complex *F. fujikuroi*. The complex of species is capable of producing a variety of valuable secondary metabolites, among them gibberellins [52, 53]. Wei and Wu [54] performed a systematic review, indicating a total of 162 new secondary metabolites, including polyketides, alkaloids, terpenes, peptides and steroids, isolated and characterized from species of *Fusarium* in the last 6 years, in which most metabolites show biological activities mean evidence the importance of carrying out investigations related to *Fusarium* species from all perspectives.

This study is the first to report the optimized production of SL by fungi from the *F. fujikuroi* complex, highlighting the strains’ potential for lipase production and detailing their biochemical properties. Opening opportunities for research on the production and application of these fungal enzymes. It achieved a hydrolytic activity of 1750 U mL ^−1^, highlighting the potential of fungal isolates in the production of SL. The lipase activity observed in this study (1750 U/mL) is significantly higher than that reported in previous studies involving species of the genus *Fusarium*. In a recent study, for example, *Fusarium solani* reached a maximum activity of 215.68 U/mL under submerged fermentation using coconut oil as substrate [55]. Although both studies used similar systems of cultivation, the enzymatic activity obtained in this study was more than eight times higher, evidencing the high biotechnological potential of the strains isolated from bacaba fruits. Similarly, Bakri et al. [16] reported lipid activities of 170 and 165 U/g dry substrate with *Fusarium culmorum* SY6, employing solid state fermentation with corn husk and tomato pulp, respectively. Despite the differences in cultivation methodologies and measurement units (U/mL vs. U/g), the significantly higher activity recorded in this study reinforces the differentiated enzymatic capacity of Amazonian strains. These comparisons highlight not only the effectiveness of the methods of isolation and cultivation employed, but also the immense yet unexplored potential of microorganisms in the Amazon rainforest. The unique biodiversity of this region may contain valuable genetic resources for the production of industrial enzymes, such as lipases. Therefore, the preservation of this biome is essential to enable future discoveries and the development of sustainable biotechnological processes.

The results obtained by the surface response to the hydrolytic activity of olive oil by the soluble lipase (Figure 3a) and its contour curves (Figure 3b), they denote an optimal zone for hydrolytic activity for pH between 5.0 and 7.0 and 31°C to 38°C for temperature. Studying the biochemical properties of enzymes, particularly their response to pH and temperature, is crucial for understanding enzymatic action. pH affects amino acid protonation, influencing protein folding, active site functionality, and enzyme stability, while temperature impacts stability, with higher temperatures often causing thermal denaturation. Sensitivity to these factors varies based on the enzyme, its source organism, and exposure duration, underscoring the intricate relationship between pH, temperature, and enzyme activity and stability [56].

Lipases are enzymes which catalyze the hydrolysis of long chain triglycerides, diglycerides, and monoglycerides into free fatty acids and glycerol, playing key roles in fungal biological pathways. Their specificity for certain fatty acids is particularly significant, as they influence the composition of lipid hydrolysis products and fatty esters produced through enzymatic synthesis, making lipases highly valuable for both theoretical research and industrial applications [57, 58].

The optimal conditions identified in this study for lipase activity—pH 5.0–7.0 and temperatures between 31°C and 38°C —are particularly advantageous for several industrial applications. This neutral–acidic pH range is ideal for the hydrolysis of waste oils in biorefineries, facilitating the breakdown of triglycerides into free fatty acids and glycerol, essential steps in biodiesel production and other bioprocesses. For example, Ribeiro et al. [59] reported that lipases from *Candida rugosa* exhibit optimal activity within a pH range of 5–8 and temperatures between 35°C and 50°C, closely aligning with the conditions observed in our study. Similarly, a lipase derived from *Acinetobacter* sp. AU07 demonstrated maximal activity at pH 7.0°C and 30°C, highlighting the efficacy of such enzymes under mild conditions suitable for industrial processes [60]. Furthermore, a cold‐adapted lipase from *Rhizomucor endophyticus* showed optimal activity at pH 6.0°C and 40°C, further emphasizing the potential of enzymes that operate efficiently at moderate temperatures and near‐neutral pH levels [61].

The strains of *Fusarium* isolated from bacaba fruits demonstrated the ability to produce lipases with optimal activity at moderate temperatures and within a range of slightly acidic to neutral pH (5.0–7.0). This pH profile contrasts with that of the lipases of other *Fusarium* species, which usually present optimal activity in more alkaline conditions. For example, *Fusarium solani* grown in submerged fermentation with coconut oil showed maximum enzymatic stability at pH 8.0°C and 35°C [55]. Similarly, Jallouli et al. [62] reported a recombinant lipase of *F. solani* expressed in Pichia pastoris with optimal activity at pH 8.0–9.0. The milder pH conditions observed in Amazonian isolates suggest a greater adaptability of their enzymes to industrial processes operating in neutral or slightly acidic environments.

The Brazilian Amazon, with its vast biodiversity and variety of fruit species, serves as a rich source of understudied fungi and yeasts with significant biotechnological value [63]. Preserving this biome is essential for future biotechnological discoveries, as it harbors a vast and largely unexplored reservoir of microbial diversity with immense potential for innovative applications, such as the development of bioproducts and environmentally efficient industrial processes. This study underscores the importance of microbial isolation and identification to enhance knowledge, promote forest preservation, and unlock the biotechnological potential of these microorganisms.

In conclusion: The fungal strains FF1 and FF2, isolated from bacaba fruits in the Brazilian Amazon, were identified as belonging to the *Fusarium fujikuroi* complex, with high similarity to *Fusarium nygamai* based on sequences from the ITS region. However, the limitation of this marker to precise identification at the species level highlights the need for complementary analyzes, integrating morphological characteristics and more informative molecular markers. This study represents the first report of the presence of *Fusarium* in bacaba fruits in the Amazon, contributing to the knowledge of regional microbial diversity and evidencing its biotechnological potential. The soluble lipase produced by these strains showed optimal activity at pH 5.0–7.0 and temperature between 31°C and 38°C, indicating possible application in industrial processes. Therefore, it is recommended to carry out pilot scale tests for the production and evaluation of lipase using substrates of industrial origin, such as palm oil residues, slaughterhouse effluents and by‐products of the food industry. These tests will allow to validate the efficiency of the process in conditions closer to industrial reality, as well as provide important data on yield, stability and possible interferences associated with the complexity of the substrates used.

## Author Contributions

**Lucely Nogueira dos Santos:** conceptualization, investigation, resources, writing – original draft, writing – review and editing, methodology. **Rafael Firmani Perna:** conceptualization, validation, resources, supervision, project administration, funding acquisition, visualization. **Ana Carolina Vieira:** validation, formal analysis, investigation, resources, writing – original draft, writing – review and editing, visualization. **Kalebe Ferreira Furtado:** investigation, writing – original draft, writing – review and editing, methodology. **Alex Fernando Almeida:** validation, formal analysis, investigation, project administration, writing – review and editing, funding acquisition. **Cristiane Angélica Ottoni:** validation, formal analysis, investigation, writing – original draft, writing – review and editing, funding acquisition, methodology, visualization, software. **Leandro Mantovani Castro:** conceptualization, validation, formal analysis, investigation, writing – original draft, writing – review and editing, methodology, software. **Felipe Mello:** validation, formal analysis, investigation, resources, writing – original draft, methodology, software. **Yoannis Domínguez:** conceptualization, validation, investigation, resources, writing – original draft, methodology, visualization, software. **Nelson Rosa Ferreira:** conceptualization, validation, formal analysis, resources, writing – original draft, writing – review and editing, supervision, project administration, funding acquisition, methodology, visualization.

## Conflicts of Interest

The authors declare no conflict of interest.

## Data Availability

Data sharing is not applicable to this article as no new data were created or analyzed in this study.

## References

[1] M. S. Afonso , L. P. N. Lopes , M. M. Ferreira , et al., “Bacaba, Pracaxi and Uxi Oils for Therapeutic Purposes: A Scoping Review,” Journal of Oleo Science 73 (2024): 11–23. 38171726 10.5650/jos.ess23142

[2] J. C. Rodrigues , S. W. Lima da , S. D. Ribeiro , et al., “Antimicrobial Activity of *Aspergillus* Sp. From the Amazon Biome: Isolation of Kojic Acid,” International Journal of Microbiology 17 (2022): 4010018. 10.1155/2022/4010018 PMC9129978 35620355

[3] M. S. P. Oliveira , A. V. Carvalho , A. F. N. Domingues , N. P. Oliveira , and E. F. Moura , Cap. 5 ‐ *Alimentícias. Espécies nativas da flora brasileira de valor econômico atual ou potencial: plantas para o futuro: região norte* (DF: MMA, 2022), 1452, ISBN 978‐65‐88265‐16‐1 (on‐line).

[4] O. V. Santos , A. A. Viana , S. D. Soares , et al., “Industrial Potential of Bacaba (*Oenocarpus bacaba*) in Powder: Antioxidant Activity, Spectroscopic and Morphological Behavior,” Food Science and Technology International 42 (2021): e62820.

[5] C. E. Willing , P. T. Pellitier , M. E. Van Nuland , et al., “A Risk Assessment Framework for the Future of Forest Microbiomes in a Changing Climate,” Nature Climate Change 14 (2024): 448–461.

[6] D. V. Wilke , P. C. Jimenez , P. C. Branco , et al., “Anticancer Potential of Compounds From the Brazilian Blue Amazon,” Planta Medica 87 (2021): 49–70. 33142347 10.1055/a-1257-8402

[7] S. Raturi and S. Kumari , “Chapter 26 ‐ Forest fungi: Advancement of White biotechnology via forest fungi, Editor(s): Editor 1, Azeem, A. M. A., Editor 2, Gryzenhout, M., Editor 3, Ghosh, S., Editor 4, Mohammed, T. A.” Forest Fungi (Academic Press, 2025), 479–488, ISBN9780443188701.

[8] V. M. Corbu , I. Gheorghe‐Barbu , A. Dumbravă , C. O. Vrâncianu , and T. E. Șesan , “Current Insights in Fungal Importance—A Comprehensive Review,” Microorganisms 11 (2023): 1384. 37374886 10.3390/microorganisms11061384 PMC10304223

[9] A. I. Adetunji and A. O. Olaniran , “Production and Potential Biotechnological Applications of Microbial Surfactants: An Overview,” Saudi Journal of Biological Sciences 28 (2021): 669–679. 33424354 10.1016/j.sjbs.2020.10.058 PMC7783833

[10] A. C. Miranda Batista , L. Nogueira dos Santos , K. Ferreira Furtado , G. C. Albuquerque Chagas Junior, Jr. , and N. Rosa Ferreira , “Análise Do Potencial Enzimático De Fungos Filamentosos Isolados Da Bacaba (*Oenocarpus bacaba*),” Amazônia Science and Health 12 (2024): 294–309.

[11] I. Todorović , Y. Moënne‐Loccoz , V. Raičević , J. Jovičić‐Petrović , and D. Muller , “Microbial Diversity in Soils Suppressive to *Fusarium* Diseases,” Frontiers in Plant Science 14 (2023): 1228749. 38111879 10.3389/fpls.2023.1228749 PMC10726057

[12] M. Ajmal , A. Hussain , A. Ali , H. Chen , and H. Lin , “Strategies for Controlling the Sporulation in *Fusarium* Spp,” Journal of Fungi 9 (2023): 10. 10.3390/jof9010010 PMC9861637 36675831

[13] P. W. Crous , L. Lombard , M. Sandoval‐Denis , et al., “ *Fusarium*: More Than a Node or a Foot‐shaped basal Cell,” Studies in Mycology 98 (2021): 100116. 34466168 10.1016/j.simyco.2021.100116 PMC8379525

[14] M. S. Vineetha , N. A. Aldabaan , S. S. More , et al., “Production and Optimization of L‐Glutaminase From Halophilic *Fusarium Solani‐Melongenae* Strain CRI 24 Under Submerged and Solid State Fermentation,” Journal of Pure and Applied Microbiology 18 (2024): 593–604.

[15] N. Marđetko , A. Trontel , M. Novak , et al., “Screening of Lignocellulolytic Enzyme Activities in Fungal Species and Sequential Solid‐State and Submerged Cultivation for the Production of Enzyme Cocktails,” Polymers 13 (2021): 3736. 34771293 10.3390/polym13213736 PMC8588072

[16] Y. Bakri , Y. Akeed , R. Ouda , T. Al‐Domani , M. Arabi , and M. Jawhar , “Lipase Production by Fusarium Culmorum in Solid State Fermentation,” Advances in Horticultural Science 29 (2015): 181–184.

[17] T. A. Thomas and S. Tirumale , “Production of a Polyketide Pigment by *Fusarium Chlamydosporum* ,” Journal of Pure and Applied Microbiology 16 (2022): 1318–1329.

[18] P. Amuzu , X. Pan , X. Hou , et al., “Recent Updates on the Secondary Metabolites From *Fusarium* Fungi and Their Biological Activities (Covering 2019 to 2024),” Journal of Fungi 10 (2024): 778. 39590697 10.3390/jof10110778 PMC11596042

[19] M. Watanabe , T. Yonezawa , K. Lee , et al., “Molecular Phylogeny of the Higher and Lower Taxonomy of the *Fusarium* Genus and Differences in the Evolutionary Histories of Multiple Genes,” BMC Evolutionary Biology 11 (2011): 322. 22047111 10.1186/1471-2148-11-322 PMC3270093

[20] A. Hathout , S. Aly , S. Bassem , A. Sahab , and N. Abo‐Sereih , “Molecular Identification and Control of Some Pathogenic *Fusarium* Species Isolated From Maize in Egypt,” Int.J. ChemTech Res 7 (2015): 44–54. ISSN: 0974‐4290.

[21] J. E. James , J. Santhanam , L. Zakaria , et al., “Morphology, Phenotype, and Molecular Identification of Clinical and Environmental *Fusarium Solani* Species Complex Isolates From Malaysia,” Journal of Fungi 8 (2022): 845. 36012833 10.3390/jof8080845 PMC9409803

[22] L. M. Teixeira , L. Coelho , and N. D. Tebaldi , “Characterization of *Fusarium Oxyporum* Isolates and Resistance of Passion Fruit Genotypes to Fusariosis,” Revista Brasileira de Fruticulture 39 (2016): e‐415.

[23] C. C. Aparecido and E. C. Rosa , “Avaliação Morfologia E Molecular Para Identificação De *Fusarium* sp,” O Biológico 81 (2019): 1–7.

[24] T. J. White , T. Bruns , S. Lee , and J. Taylor , “Amplification and direct sequencing of fungal ribosomal RNA genes for phylogenetics.” in PCR Protocols: A guide to methods and applications, eds. M. Innis , D. Gelfand , J. Sninsky and T. White (San Diego: Academic Press, 1999), 315–322.

[25] T. A. Hall , “Bioedit: A User‐Friendly Biological Sequence Alignment Editor and Analysis Program for Windows 95/98/NT. (v7.0.9.0. 2007),” Nucleic Acids Symposium Series 41 (1999): 95–98.

[26] D. A. Benson , M. Cavanaugh , K. Clark , et al., “Genbank,” Nucleic Acids Research 46, no. D1 (2018): D41–D47. 29140468 10.1093/nar/gkx1094 PMC5753231

[27] Flora and Fungi of Brazil. *Rio de Janeiro Botanical Garden* . Available at: http://floradobrasil.jbrj.gov.br/, accessed on: 24 May 2024.

[28] K. Katoh , J. Rozewicki , and K. D. Yamada , “Mafft Online Service: Multiple Sequence Alignment, Interactive Sequence Choice and Visualization,” Briefings in Bioinformatics 20 (2019): 1160–1166. 28968734 10.1093/bib/bbx108 PMC6781576

[29] F. Ronquist , M. Teslenko , P. van der Mark , et al., “Mrbayes 3.2: Efficient Bayesian Phylogenetic Inference and Model Choice Across a Large Model Space,” Systematic Biology 61 (2012): 539–542. 22357727 10.1093/sysbio/sys029 PMC3329765

[30] F. Ronquist and J. P. Huelsenbeck , “Mrbayes 3: Bayesian Phylogenetic Inference Undermixed Models,” Bioinformatics 19 (2003): 1572–1574. 12912839 10.1093/bioinformatics/btg180

[31] S. Guindon and O. Gascuel , “A Simple, Fast, and Accurate Algorithm to Estimate Large Phylogenies by Maximum Likelihood,” Systematic Biology 52 (2003): 696–704. 14530136 10.1080/10635150390235520

[32] D. Darriba , G. L. Taboada , R. Doallo , and D. Posada , “Jmodeltest 2: More Models, New Heuristics and Parallel Computing,” Nature Methods 9 (2012): 772. 10.1038/nmeth.2109 PMC4594756 22847109

[33] H. Akaike , “Information theory and an extension of the maximum likelihood principle.” in Second international symposium on information theory, eds. B. N. Petrov and F. Csaki (Budapest, Hungary: Akadémiai Kiadó, 1973), 267–281).

[34] W. K. Hastings , “Monte Carlo Sampling Methods Using Markov Chains and Their Applications,” Biometrika 57 (1970): 97–109.

[35] A. Rambaut FigTree version 1.4. Tree Figure Drawing Tool. Institute of Evolutionary Biology. University of Edinburg. 2018. http://tree.bio.ed.ac.uk/software/figtree/, accessed 29 Jun 2024.

[36] F. V. Diniz , Y. M. M. Lima , F. S. Paz , et al., “Atividade Enzimática De Fungos Endofíticos De Bacaba (*Oenocarpus bacaba* Mart.),” Biota Amazônia. Macapá 10 (2020): 7–11.

[37] A. F. De Almeida , S. M. Taulk‐Tornisielo , and E. C. Carmona , “Influence of Carbon and Nitrogen Sources on Lipase Production by a Newly Isolated *Candida viswanathii* Strain,” Annals of Microbiology 63 (2013): 1225–1234.

[38] C. M. F. Soares , H. F. De Castro , F. F. De Moraes , and G. M. Zanin , “Characterization and Utilization of *Candida rugosa* Lipase Immobilized on Controlled Pore Silica,” Applied Biochemistry and Biotechnology 79 (1999): 745–758. 10.1385/abab:79:1-3:745 15304694

[39] G. Kuang , Y. D. U , S. Lu , et al., “Silica@Lipase Hybrid Biocatalysts With Superior Activity by Mimetic Biomineralization in Oil/Water Two‐Phase System for Hydrolysis of Soybean Oil,” LWT 160 (2022): 113333.

[40] A. F. Siegel and M. R. Wagner , “Chapter 12 ‐ Multiple Regression: Predicting One Variable from Several Others.” in Practical Business Statistics (Sixth Edition), eds. F. Siegel Andrew . Academic Press, 2012), 347–416.

[41] R. Patel , K. Mehta , J. Prajapati , et al., “An Anecdote of Mechanics for Fusarium Biocontrol by Plant Growth Promoting Microbes,” Biological Control 174 (2022): 105012.

[42] P. D. da Rosa , V. Aquino , A. M. Fuentefria , and L. Z. Goldani , “Diversity of Fusarium Species Causing Invasive and Disseminated Infections,” Journal of Medical Mycology 31 (2021): 101137. 33932878 10.1016/j.mycmed.2021.101137

[43] M. Torbati , M. Arzanlou , and A. C. da Silva Santos , “Fungicolous Fusarium Species: Ecology, Diversity, Isolation, and Identification,” Current Microbiology 78 (2021): 2850–2859. 34184111 10.1007/s00284-021-02584-9

[44] R. Landeweert , P. Leeflang , T. W. Kuyper , et al., “Molecular Identification of Ectomycor‐Rhizal Mycelium In Soil Horizons,” Applied and Environmental Microbiology 69 (2003): 327–333. 12514012 10.1128/AEM.69.1.327-333.2003 PMC152382

[45] S. M. Murodova , T. A. Bozorov , I. S. Aytenov , et al., “Uncovering *Fusarium* Species Associated With *Fusarium* Wilt in Chickpeas (*Cicer Arietinum L*.) and the Identification of Significant Marker–Trait Associations for Resistance in the International Center for Agricultural Research in the Dry Areas' Chickpea Collection Using SSR Markers,” Agronomy 14 (1943): 2024.

[46] J. Harish , P. P. Jambhulkar , R. Bajpai , et al., “Morphological Characterization, Pathogenicity Screening, and Molecular Identification of *Fusarium* Spp. Isolates Causing Post‐Flowering Stalk Rot in Maize,” Frontiers in Microbiology 14 (2023): 1121781. 37065162 10.3389/fmicb.2023.1121781 PMC10102488

[47] D. M. Geiser , M. Del mar jiménez‐Gasco , S. Kang , et al., “ *FUSARIUM*‐ID v. 1.0: A DNA sequence database for identifying *Fusarium* ,” European Journal of Plant Pathology 110 (2004): 473–479.

[48] P. W. Crous , L. Lombard , M. Sandoval‐Denis , et al., “ *Fusarium*: more than a node or a foot‐shaped basal Cell,” Studies in Mycology 98 (2021, 17): 100116. 34466168 10.1016/j.simyco.2021.100116 PMC8379525

[49] S. Janevska and B. Tudzynski , “Secondary Metabolism in *Fusarium fujikuroi*: Strategies to Unravel the Function of Biosynthetic Pathways,” Applied Microbiology and Biotechnology 102 (2018): 615–630. 29204899 10.1007/s00253-017-8679-5

[50] N. Yilmaz , M. Sandoval‐Denis , L. Lombard , C. M. Visagie , B. D. Wingfield , and P. W. Crous , “Redefining Species Limits in the *Fusarium fujikuroi* Species Complex,” Persoonia 46 (2021): 129–162. 35935895 10.3767/persoonia.2021.46.05 PMC9311392

[51] M. Zhang , C. Peng , S. Li , and C. Tian , “Morphological and Phylogenetic Analyses Reveal Two New Species of the *Fusarium fujikuroi* (*Hypocreales, Nectriaceae*) Species Complex in China,” MycoKeys 112 (2025): 127–163. 39867689 10.3897/mycokeys.112.133472 PMC11758097

[52] Y. K. Cen , J. G. Lin , Y. L. Wang , J. Y. Wang , Z. Q. Liu , and Y. G. Zheng , “The Gibberellin Producer *Fusarium fujikuroi*: Methods and Technologies in the Current Toolkit,” Frontiers in Bioengineering and Biotechnology 8 (2020): 232. 32292777 10.3389/fbioe.2020.00232 PMC7118215

[53] J. B. Pandya , A. N. Patani , V. H. Raval , K. N. Rajput , and R. R. Panchal , “Understanding the Fermentation Potentiality for Gibberellic Acid (GA3) Production Using Fungi,” Current Microbiology 80 (2023): 385. 37874373 10.1007/s00284-023-03454-2

[54] J. Wei and B. Wu , “Chemistry and Bioactivities of Secondary Metabolites From the Genus *Fusarium* ,” Fitoterapia 146 (2020): 104638. 32585294 10.1016/j.fitote.2020.104638

[55] A. K. de Carvalho Silva , F. J. L. Lima , K. R. A. Borges , et al., “Utilization of Fusarium Solani Lipase for Enrichment of Polyunsaturated Omega‐3 Fatty Acids,” Brazilian Journal of Microbiology 55 (2024): 2211–2226. 38874742 10.1007/s42770-024-01411-0 PMC11405586

[56] M. F. Kabir and L. K. Ju , “On Optimization of Enzymatic Processes: Temperature Effects on Activity and Long‐Term Deactivation Kinetics,” Process Biochemistry 130 (2023): 734–746.

[57] S. Kim , J. Lee , J. Park , et al., “Genetic and Transcriptional Regulatory Mechanisms of Lipase Activity in the Plant Pathogenic Fungus *Fusarium graminearum* ,” Microbiology Spectrum 11 (2023): e0528522. 37093014 10.1128/spectrum.05285-22 PMC10269793

[58] K. A. Baloch , A. Singh , K. Pudtikajorn , and S. Benjakul , “Lipases From Different Yeast Strains: Production and Application for n‐3 Fatty Acid Enrichment of Tuna Eyeball Oil,” Biocatalysis and Agricultural Biotechnology 48 (2023): 102651.

[59] B. D. Ribeiro , A. M. de Castro , M. A. Coelho , and D. M. Freire , “Production and Use of Lipases in Bioenergy: A Review From the Feedstocks to Biodiesel Production,” Enzyme Research 2011 (2011): 615803. 21785707 10.4061/2011/615803 PMC3137985

[60] P. Gururaj , S. Ramalingam , G. Nandhini Devi , and P. Gautam , “Process Optimization for Production and Purification of a Thermostable, Organic Solvent Tolerant Lipase From *Acinetobacter* Sp. Au07,” Brazilian Journal of Microbiology 47 (2016): 647–657. 27268114 10.1016/j.bjm.2015.04.002 PMC4927683

[61] Q. Yan , X. Duan , Y. Liu , Z. Jiang , and S. Yang , “Expression and Characterization of a Novel 1,3‐regioselective Cold‐Adapted Lipase From *Rhizomucor endophyticus* Suitable for Biodiesel Synthesis,” Biotechnology for Biofuels 9 (2016): 86. 27081399 10.1186/s13068-016-0501-6 PMC4831154

[62] R. Jallouli , G. Parsiegla , F. Carrière , Y. Gargouri , and S. Bezzine , “Efficient Heterologous Expression of *Fusarium solani* Lipase, FSL2, in Pichia Pastoris, Functional Characterization of the Recombinant Enzyme and Molecular Modeling,” International Journal of Biological Macromolecules 94 (2017): 61–71. 27620466 10.1016/j.ijbiomac.2016.09.030

[63] A. C. M. Lima , I. L. S. Lunara , T. A. Bastos , F. C. Paula‐Elias , and A. F. Almeida , Potencial das frutas amazônicas para a produção de enzimas microbiana. Chap 4. Frutos Amazônicos: Biotecnologia e Sustentabilidade, eds. A. F. de Almeida; and C. C. A. A. Santos . e‐Book. Palmas, TO: EDUFT, 2020. ISBN:978‐65‐89119‐14‐2).

